# Controversies about the visual areas located at the anterior border of area V2 in primates

**DOI:** 10.1017/S0952523815000188

**Published:** 2015-10-20

**Authors:** RICARDO GATTASS, BRUSS LIMA, JULIANA G.M. SOARES, LESLIE G. UNGERLEIDER

**Affiliations:** 1 Laboratory of Cognitive Physiology, Institute of Biophysics Carlos Chagas Filho, UFRJ, Rio de Janeiro, RJ 21941-900, Brazil; 2 Laboratory of Brain and Cognition, National Institute of Mental Health, National Institutes of Health, Bethesda, Maryland 20892

**Keywords:** Visual system, Extrastriate cortex, V3, V4, PO, POd, V6

## Abstract

Anatomical and electrophysiological studies have provided us with detailed information regarding the extent and topography of the primary (V1) and secondary (V2) visual areas in primates. The consensus about the V1 and V2 maps, however, is in sharp contrast with controversies regarding the organization of the cortical areas lying immediately rostral to V2. In this review, we address the contentious issue of the extent of the third visual area (V3). Specifically, we will argue for the existence of both ventral (V3v) and dorsal (V3d) segments of V3, which are located, respectively, adjacent to the anterior border of ventral and dorsal V2. V3v and V3d would together constitute a single functional area with a complete representation of both upper and lower visual hemifields. Another contentious issue is the organization of the parietal-occipital (PO) area, which also borders the rostral edge of the medial portion of dorsal V2. Different from V1, V2, and V3, which exhibit a topography based on the defined lines of isoeccentricity and isopolar representation, area PO only has a systematic representation of polar angles, with an emphasis on the peripheral visual field (isoeccentricity lines are not well defined). Based on the connectivity patterns of area PO with distinct cytochrome oxidase modules in V2, we propose a subdivision of the dorsal stream of visual information processing into lateral and medial domains. In this model, area PO constitutes the first processing instance of the dorsal-medial stream, coding for the full-field flow of visual cues during navigation. Finally, we compare our findings with those in other species of Old and New World monkeys and argue that larger animals, such as macaque and capuchin monkeys, have similar organizations of the areas rostral to V2, which is different from that in smaller New World monkeys.

## Introduction

Talbot and Marshall (1940) proposed a breakthrough concept in cortical organization, by demonstrating that the retinal surface (and, consequently, the visual field) was topographically represented on the surface of the occipital lobe. A couple of decades later Daniel and Whitteridge (1961) extended this finding to demonstrate a complete representation of the visual field in the primary visual cortex of the macaque. However, it was the work of Cowey (1964) that provided the first glimpse of what we may still take for granted today; namely, that the visual field is represented multiple times in the cortex, in distinct visual areas. Indeed, techniques as diverse as anatomical tracing and brain imaging have provided additional evidence for the existence of multiple, topographically organized visual areas, each containing a complete or partial representation of the visual field (reviewed in Gattass et al., 2005).

In this scenario, two visual areas have emerged rather undisputed. They are the first (V1) and second (V2) visual areas, each exhibiting a precise topographic representation of the contralateral visual field (Allman & Kaas, 1974; Gattass et al., 1981, 1987, 1997; Rosa et al., 1988*b*). Despite this fact, we are surrounded by controversies regarding the organization of certain areas located rostral to the V2 border, particularly at its dorsal segment. This debate is important, among other reasons, due to the longstanding proposal that visual information, subsequent to its processing in areas V1 and V2, flows through parallel pathways, namely the dorsal and ventral streams of visual processing (Ungerleider & Mishkin, 1982). A thorough understanding of the cortical organization immediately rostral to V2 will potentially elucidate how visual information is partitioned before its full segregation into separate streams of information flow.

**Fig. 1.**
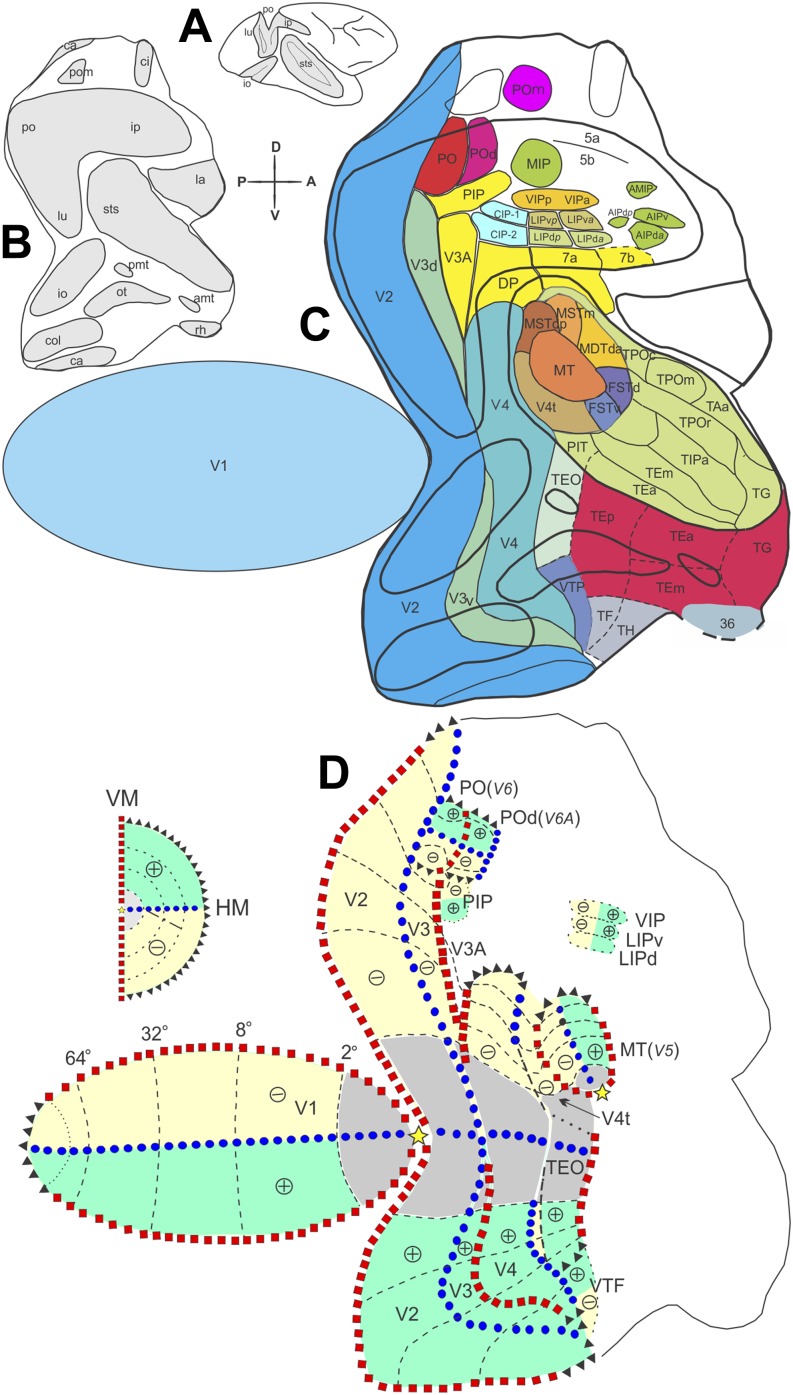
Posterior visual areas exhibit an organized topographic organization. Two-dimensional reconstruction of the monkey cortex, showing the location of the striate and extrastriate visual areas found in the macaque and capuchin monkeys. The right hemisphere (**A**), shown with opened sulci, underwent a physical flattening procedure (**B** and **C**). Different colors label the different areas. The gray regions in (**A**) and (**B**) indicate the cortex buried within the sulci. Note that the flattened map of V1 was separated from the extrastriate cortex during the physical flattening procedure (**C**). Heavy lines indicate the boundaries of the sulci; thin lines indicate the boundaries between visual, visuomotor, and polissensory areas. The dashed lines indicate the boundaries of the temporal areas defined in the macaque, based on cortical connections. The dotted-dashed lines indicate the boundaries between the neocortex and allocortex. (**D**) Visuotopic organization of the cortical visual areas shown on a two-dimensional reconstruction of the monkey cortex. The VM is represented by red squares, the HM by blue circles, the eccentricity lines by dashed lines, the visual field periphery by black triangles, the foveal region by light gray and the fovea by yellow stars. + and − indicate the upper and lower visual quadrants, respectively, which are labeled in light green and yellow. The insert illustrates the contralateral visual hemifield. For the names of areas, see *Abbreviations*.

Area middle temporal (MT), situated further anteriorly, has a well-established location, extent and topographic organization, in both Old and New World monkeys (Allman & Kaas, 1971; Gattass & Gross, 1981; Fiorani et al., 1989; Rosa et al., 1997; Rosa & Elston, 1998). Curiously, the cortical organization in between V2 and MT remains highly disputed. There are several possible reasons for this longstanding lack of agreement. First, there could be significant differences in the cortical organization of this region when comparing animals of different brain and body sizes (Chaplin et al., 2013) or, alternatively, gyrencephalic (Old World and certain large New World monkeys) and lissencephalic monkeys (small New World monkeys). Second, the presence of the lunate sulcus (and the corresponding annectent gyrus at its fundus) in species, such as the macaque and capuchin monkeys, hinders clear myeloarchitectonic delimitation of the areas in the disputed territory. Third, some of the areas anterior to V1 have split representations of the upper and lower visual fields, which are located, respectively, in ventral and dorsal portions of the occipital cortex (e.g., area V2, see Fig. 1). Furthermore, it is possible in some cases that the ventral and dorsal cortical segments, instead of forming a contiguous visual area (Sousa et al., 1991), are displaced in separate cortical islands (Fig. 1), as it has been proposed for area V3 (Gattass et al., 1988). These features of cortical organization have made it difficult to reach a consensus regarding the topographic layout of areas anterior to V2. In such circumstances, it has been tempting to attribute to certain visual areas the exclusive representation of either the lower or the upper visual hemifield (Rosa et al., 2000). Notwithstanding the risk of advancing a teleological argument, we tend to resist the temptation until exhaustive experimental evidence proves otherwise. This is because we believe that neuronal operations which are performed only on inputs arising from the lower or upper parts of the retina would result in a sharp functional or perceptual transition between hemifield quadrants.

In this review, we will focus on two of the proposed visual areas located along the rostral border of V2, namely the third (V3) and parieto-occipital (PO) areas. We will argue in favor of the existence of a V3 that includes both ventral (V3v, upper contralateral quadrant representation) and dorsal (V3d, lower contralateral quadrant representation) components in Old World macaques and New World capuchin monkeys. Accordingly, V3v and V3d are proposed to constitute a single visual area representing the entire contralateral visual fields in each cerebral hemisphere.

Evidence supporting the above model first originated from anatomical tracing studies showing that injections in V2v and V2d project, in a topographically organized manner, to ventral and dorsal cortical regions, respectively, located immediately rostral to V2 (Gattass et al., 1997). Additionally, injections throughout the extent of V4 show a similar pattern of feedback projections to the same region anterior to V2 (Ungerleider et al., 2008). We will also discuss electrophysiological data that have been controversial regarding the organization of V3d depending on the species and, in the case of the macaque, on the individual. We will speculate on how this diversity might reconcile conflicting models proposed for Old World and small New World monkeys.

Finally, we will examine the organization of area PO, and some of its neighboring areas (parieto-occipital dorsal area, POd, and parieto-occipital medial area, POm), which are located on the medial cortex near the occipital-parietal junction. As illustrated in Fig. 1, in our proposal area PO borders V2 and the medial portion of area V3d, such that V2 shares the horizontal meridian (HM) with both V3d and area PO. Here also, initial evidence implicating PO as a distinct cortical area came from tracing studies in the macaque (Colby et al., 1988; Gattass et al., 1988; Ungerleider et al., 2008). Notably, we showed that the projections from area V2 and V4 to area PO concern mainly the peripheral (>20 deg) representation of the visual field, despite the fact that some receptive fields (RFs) mapped in area PO are large enough to encompass the foveal and parafoveal representations. We will review evidence based on myeloarchitecture, cytoarchitecture and densely spaced tracer injections to emphasize that PO constitutes a single and unique visual area. We believe that the evidence available refutes the notion that PO constitutes the peripheral representation of V3d. Similarly, it equally refutes the proposal that PO is part of a larger area V6, as proposed by Galletti et al. (1999*a*, 2001). In the latter, PO would represent the peripheral visual field of V6, while the foveal representation would be contained outside PO, in the annectent gyrus.

Along our review, we will compare the third tier cortical areas between two groups of monkeys. The first group includes the Old World macaque and the large New World capuchin monkeys, while the second group encompasses the small New World Monkeys (e.g., the owl and marmoset monkeys). Our underlying hypothesis is that the macaque and the capuchin both retained, along their evolution, similar features to those present in their common ancestor, such as gyrencephaly. On the other hand, we believe that the small New World monkeys trailed a different evolutionary path, and thereby evolved a divergent set of anatomical characteristics, an example being their loss of gyrencephaly (Kelava et al., 2012, 2013). The presence of sulci and gyri is particularly relevant here. Areas immediately anterior to V2 often find themselves buried inside the lunate or the PO cleft. A transition to lissencephaly may have triggered a critical reorganization of the third tier visual areas. Finding homologies between the gyrencephalic and lissencephalic groups can potentially provide insights into the functional role of these areas, despite the fact that clear cut homologies have remained contentious.

## Cortical visual maps

In the visual system, cortical visual areas can be mapped using a variety of methods. Systematic electrophysiological techniques have revealed cortical areas with partial or complete organized maps of the visual field, with different emphasis on the central or peripheral representations (reviewed in Gattass et al., 2005). Fig. 1 shows the location of the visual areas described so far using as template the brain of a macaque or capuchin monkey; because the organization in these species is similar, according to our studies, this figure represents a “generic” summary that applies to both species. In addition to V1, V2, and V3, the figure summarizes the visual topographies of the fourth visual area (V4), of two areas located in the occipito-temporal transition [cytoarchitectural area TEO of von Bonin and Bailey 1947, and the temporal ventral posterior area (TVP), which overlaps with cytoarchitectural area TF], of motion-sensitive areas within the superior temporal sulcus (MT, its adjacent dorsal zone—V4t or DZ, and the medial superior temporal area—MST) and of areas PO and POd, located in the anterior bank of the PO sulcus [These overlap with areas “V6” and “V6A” recognized by other groups (e.g., Galletti et al., 1996, 1999*a*,*b*)].

## Anisotropies in the visual maps

Most cortical topographic maps show marked emphases on specific regions of the sensory surface. In the visual cortex, this is usually in the form of a magnified representation of the fovea, and gradual decrease in the cortical magnification factor (CMF) toward the representation of the retinal periphery (Daniel & Whitteridge, 1961; Gattass & Gross, 1981; Gattass et al., 1981; Gattass et al., 1987; Rosa et al., 1988*b*). Superimposed in these gradients is another type of irregularity, anisotropy. In brief, the CMF at a specific point of the map varies depending on the direction along which a measurement is performed; this typically results in the map being “stretched” in one direction, in comparison with the hypothetical situation in which the CMF is defined solely by the eccentricity of RFs recorded therein (Gattass et al., 1987; Rosa et al., 1988*b*). We have used anisotropy measurements to make distinctions between V1, V2, V3, and PO. In both V1 and V2, we have observed that, at any given eccentricity, the CMF is larger when measured between points located along an isopolar line (i.e., between cells with RFs at different eccentricities, but sharing a same polar angle), rather than along an isoeccentric line. We have also noticed that the anisotropy in visual representation is less pronounced in V1 than in V2, where the isopolar CMF is usually 50% higher than the isoeccentric one (Gattass et al., 1987; Rosa et al., 1988*b*). More recently, we have been studying the regularity of visual maps in other areas of the capuchin monkey. For example, in V3v, the anisotropy is even more pronounced than that in V2, with the CMF estimated along isopolar lines being at least twice that measured along isoeccentricity lines (Rosa et al., 2000).

**Fig. 2.**
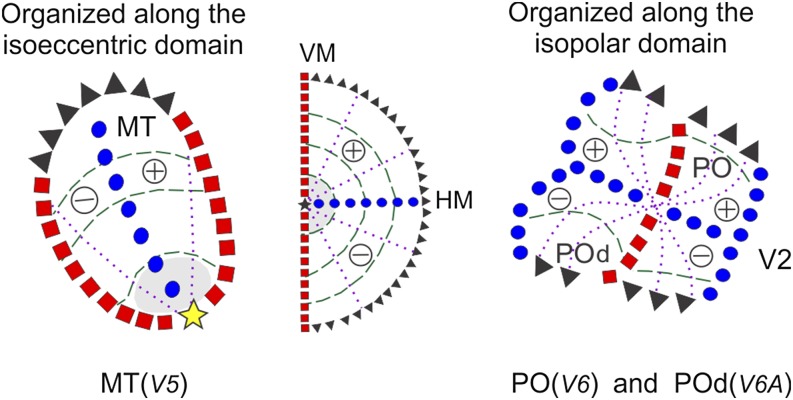
Different types of visuotopic organization based on isoeccentric and isopolar domains. Examples of visuotopic organizations with a preponderance of isoeccentric (MT) or isopolar domain representation (PO and POd). Conventions as in Fig. 1.

In areas PO and POd of the dorsomedial pathway (Gattass et al., 1990; Neuenschwander et al., 1994; Nascimento-Silva et al., 2003, 2014), we found a different type of irregularity: it is always possible to define the representation of isopolar lines but not those of the isoeccentric lines. At present, we have no direct evidence regarding the functional significance of the observed isopolar order *versus* disorder in the isoeccentric domain in these two areas. One may speculate that centrifugal and centripetal organizations of directionality, such as those observed in higher-order posterior parietal areas (which are connected to PO), demand interactions between neurons that analyze the regions of space sharing a similar polar angle but at different eccentricities. It may be that the intermixing of RF eccentricities in adjacent columns of areas PO and POd allows these interactions to occur within local circuits. This arrangement could be considered an atypical visuotopic map (Fig. 2) due to its dissimilarities with neighboring V1, V2, and V3.

## Area V2 and its connectivity

In macaques, the major cortical projection target of area V1 is area V2 (Kuypers et al., 1965; Cragg & Ainsworth, 1969; Zeki, 1969, 1971, 1976; Zeki & Sandeman, 1976; Rockland & Pandya, 1979, 1981; Lund et al., 1981; Weller & Kaas, 1985; Van Essen et al., 1986). V2 is located within area 18 of Brodmann and corresponds to area OB of von Bonin and Bailey (1947). Our group was the first to systematically map the entire extent of projection targets of V2, by injecting anterograde tracers in this area (as opposed to using retrograde traces in other areas, which receive V2 projections). In the macaque, we found that all V2 sites project topographically back to V1 and forward to V3, V4, and MT (Gattass et al., 1997). There is also a topographically organized projection from V2 to V4t, but this projection is limited to the lower visual field representation. Thus, V2 appears to project to virtually all the visual cortex within the occipital lobe. In addition to these projections to occipital visual areas, V2 sites representing eccentricities of about 30 deg and greater project to three visual areas in dorsal extrastriate cortex, namely, the MST, the PO, and the ventral intraparietal (VIP) areas.

The second visual area, when stained for the mitochondrial enzyme cytochrome oxydase (CytOx), shows a pattern of alternating thick and thin CytOx-rich stripes, running perpendicular to the V1/V2 border, separated by CytOx-poor interstripes regions (Livingstone & Hubel, 1982). These three types of stripes differ in their neuronal properties and connections (DeYoe & Van Essen, 1985; Zeki & Shipp, 1989; Roe & Ts'o, 1995; Levitt et al., 1994; Olavarria & Van Essen, 1997). In capuchin monkey V2, histological sections tangential to the cortical surface stained for CytOx revealed a pattern similar to the one described in the *Saimiri* and *Macaca* (Livingstone & Hubel, 1982; Wong-Riley & Caroll, 1984; DeYoe & Van Essen, 1985; Shipp & Zeki, 1985; Gattass et al., 1987; Rosa et al., 1988*a*; Zeki & Shipp, 1989). Thus, to evaluate whether there is a segregation of the streams of visual information processing in CytOx-modules of V2, we injected fluorescent retrograde tracers into V4, MT, and PO and studied the distribution of the labeled cells in these modules (Nascimento-Silva et al., 2014). The distribution of labeled cells provides evidence for three streams of visual processing with origins in different CytOx-modules in area V2. The data thus support the subdivision of the dorsal stream of visual information processing into a dorsomedial and a dorsolateral component (Nascimento-Silva et al., 2003, 2014), as originally proposed by Gattass et al. (1990). Within this scheme, PO would constitute the first processing stage of the dorsal-medial stream, coding for the full-field flow of visual cues during navigation.

## Area V3 in the Old World macaque monkey

**Fig. 3.**
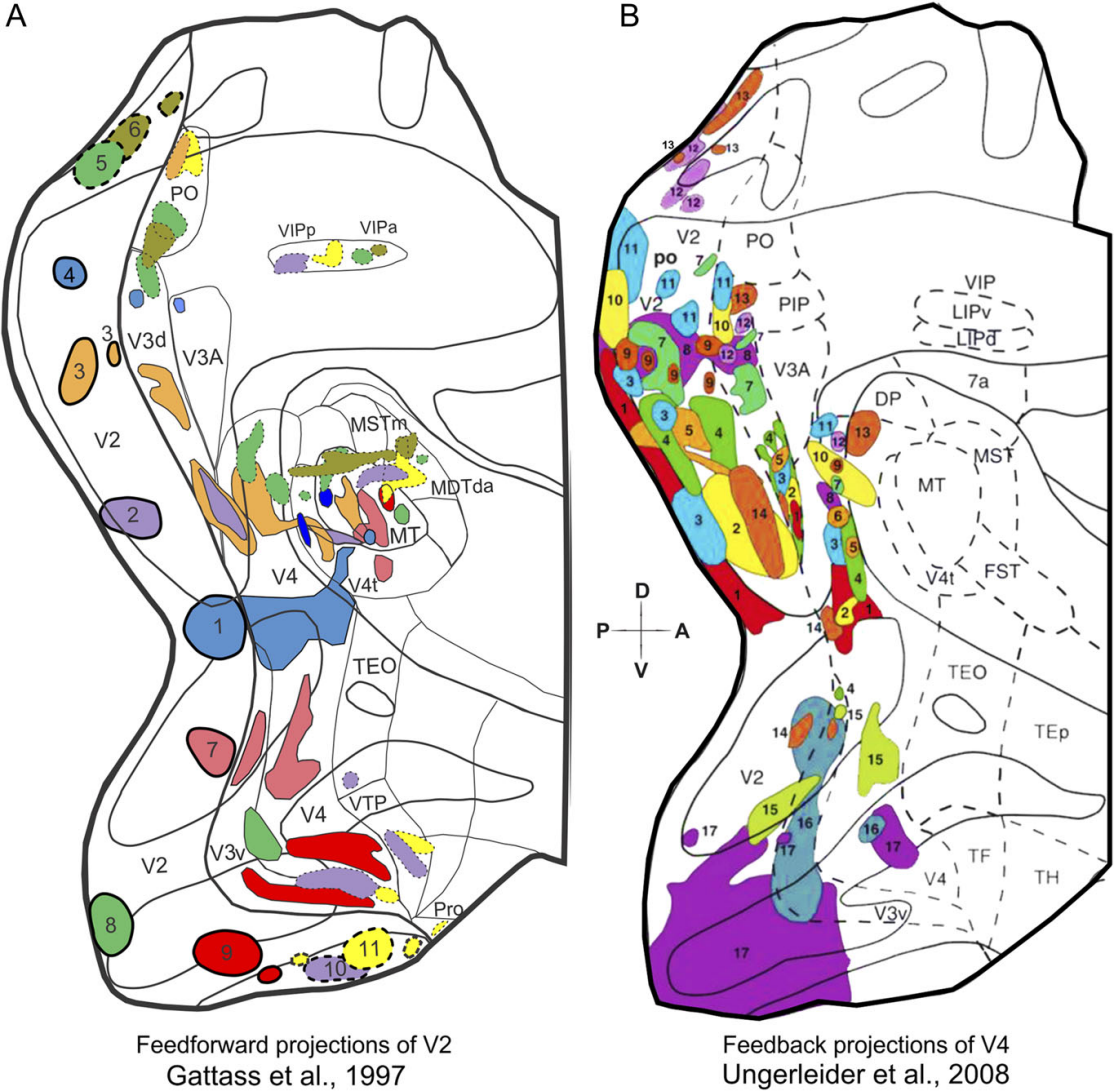
Connectivity studies support the existence of a dorsal and a ventral V3, anterior to V2. Summary maps of the V2 feedforward (**A**) and the V4 feedback (**B**) projections in the extrastriate cortex. Two-dimensional reconstruction of the macaque monkey cortex, showing the location of the extrastriate visual areas delimited by myeloarchitectonic borders. Injection and projection sites are labeled with the same color to aid visualization. (**A**) Injection sites (1–11) are plotted with a thick black outline, while their corresponding projections sites are plotted with the same color, but thin outline. Blobs with dotted outline correspond to injection or projection sites in the peripheral representation. Adapted from Gattass et al. (1997). (**B**) The corresponding injection and projections sites are labeled with the same number and color. To avoid clutter, all blobs are plotted with thin outlines. Adapted from Ungerleider et al. (2008). Please refer to Fig. 1 concerning the topographic organization of the various areas and other conventions.

The existence of a representation of the upper visual quadrant adjacent to that of area V2, in the ventral prestriate cortex, was first suggested by Cragg and Ainsworth (1969) and Zeki (1969) after anatomical tracing experiments in Old World (macaque) monkeys. This region was considered to be part of a “third visual area” (V3), which wrapped around V2 both dorsally and ventrally. However, there are currently two different views regarding the organization of V3 in macaques. Based on electrophysiological mapping studies, Gattass and colleagues (Gattass et al., 1988) have argued that the entire region bordering V2 anteriorly is a single visual area which contains a representation out to 30–40 deg eccentricity in both the upper (V3v) and lower (V3d) visual fields. Gattass et al. (1997) demonstrated that, although V1 may project asymmetrically to V3, V2 does not. Whereas the upper field representation of V2 projects to V3v, the lower field representation of V2 projects to V3d. The results from the feedforward projection studies in dorsal and ventral V2 serve to delineate the dorsal and ventral portions of V3. H^3^ amino acids injections in V2 (Fig. 3A) demonstrate that this area projects topographically back to V1 (data not shown) and forward to V3, V4, and MT. Peripheral injections in V2 project to both V3d, as well as to PO (Sousa et al., 1991; Gattass et al., 1997). Note, for example, that injections number 9, 8, 7, 1, 2, 3, and 4 situated, respectively, along the ventral to dorsal extension of V2 (see Fig. 3A), project in an orderly fashion to the strip of cortex immediately anterior to it (i.e., V3v and V3d, respectively).

Injections of anterograde and retrograde tracers in different topographical locations of V4 are also capable of revealing the borders of dorsal and ventral V3. Central injections in V4 (Fig. 3B) showed feedback projections to V3 both dorsally and ventrally, while far peripheral injections in V4 resulted in projections to V3v, V3d, and PO (Ungerleider et al., 2008). Note that injections number 17, 16, 15, 1, 2, 3, 7, 10, 12, 13, 11 placed along V4 (see Fig. 3B) project to V3 following the same pattern as described for V2 above. Altogether, densely spaced tracer injections indicate that V2 and V4 send topographically organized projections to both V3v and V3d, suggesting that these two segments are part of a single and homogeneous area. Our projection results also show that the visual field representation within V3 may extend beyond 40 deg eccentricity, but it does not extend all the way out to 80 deg. Thus, there is a reduction in the extent of cortical visual field representation as one moves from V2 to V3, a finding that is consistent with our electrophysiological findings (Gattass et al., 1981, 1988, 2005). Finally, the anatomical results show that V3d shares the representation of the vertical meridian (VM) with areas V4 and PO, which is again consistent with our electrophysiological findings (Gattass et al., 1985, 1988).

By contrast, several authors have argued that the cortex anterior to V2, dorsally and ventrally, contains different visual areas, based on differences in projections from V1, myeloarchitecture, and neural response properties (Burkhalter et al., 1986; Newsome et al., 1986; Van Essen et al., 1986; Felleman & Van Essen, 1987). These investigators have termed the upper and lower visual field representations anterior to V2 as areas VP and V3, respectively. The name VP was coined on the basis of an anatomical study of interhemispheric connections in the New World (owl) monkey, which observed a conspicuous strip of callosal terminals in the ventral cortex anterior to V2 (Newsome & Allman 1980). A similar band of callosal terminations was observed in the ventral cortex of Old World monkeys (Van Essen et al., 1982; Boussaoud et al., 1991), supporting a similar organization in both groups of simians.

**Fig. 4.**
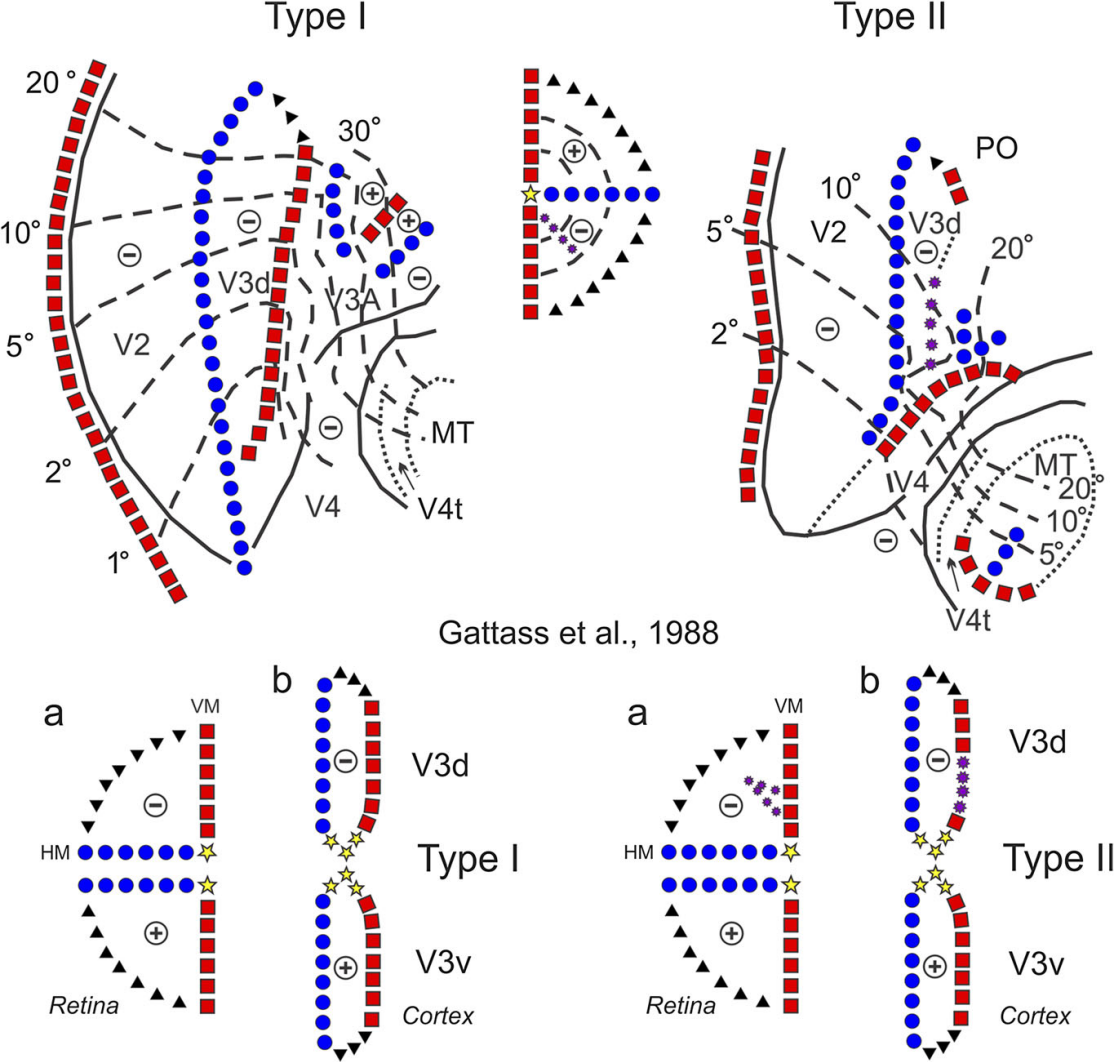
Visuotopic organization of area V3 varies across individuals. The Type I (left) and the Type II (right) variants of dorsal V3 organization described in the *Macaca fascicularis*. See text for details. Adapted from Gattass et al. (1988). Conventions as in Fig. 1.

In the Gattass et al. (1988) study of visual topography, we reported two types of organization for V3d in *Macaca fascicularis*. Fig. 4 shows the visual map of dorsal V3 in the two types of animals, and a pictorial diagram showing the hypothetical transformations (splits) in the representation of the visual field in the two V3 variants. In one type (Type I animal), the anterior border of V3 was simply the representation of the lower VM. In the other type (Type II animal), the lower VM representation was split into two segments, with a portion of the lower visual field representation lying in between. It is important to emphasize, however, that Type II animals were an exception to the rule. Type I animals, where V3 showed a continuous and smooth anterior border, were the most common variant observed.

**Fig. 5.**
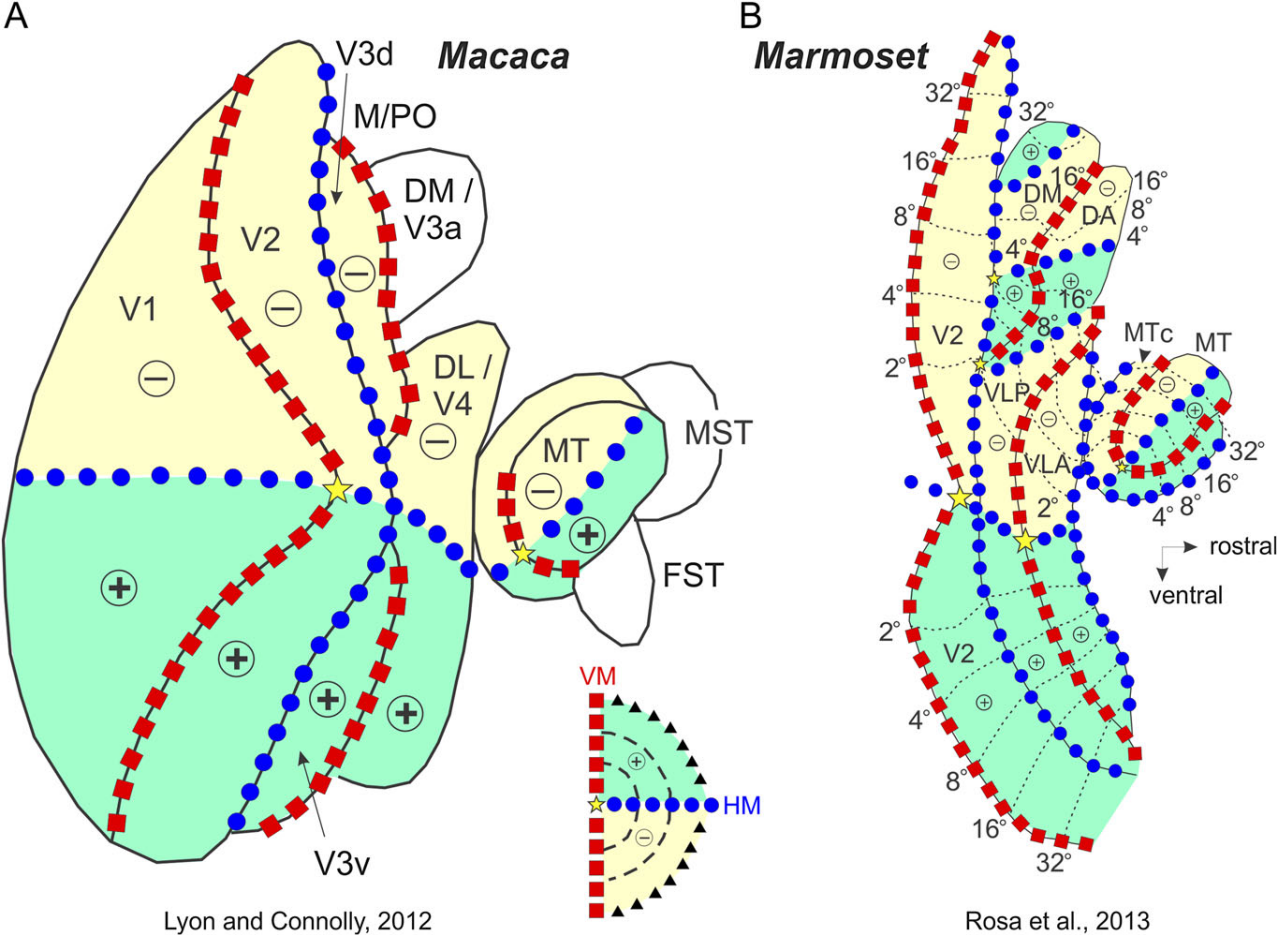
Visual cortical organization in gyrencephalic and lissencephalic monkeys diverged in a significant way along evolution. (**A**) Visuotopic organization proposed for the cortical visual areas anterior to V2 in the macaque monkey. Adapted from Lyon and Connolly (2012). (**B**) The corresponding organization proposed for small New World monkeys (marmoset). Adapted from Rosa et al. (2013). In the marmoset, VLP would correspond to V3v, and VLA to V4v. VLA is equivalent to part of DL. Conventions as in Fig. 1.

Recently, Lyon and Connolly (2012) concluded that evidence obtained in several primate species supports the hypothesis that an elongated V3 area, forming a complete map of the visual field, occupies the region anterior to V2. One central prediction of Lyon & Connolly's scheme is that cells in the dorsal half of V3 always have RFs representing the lower half of the visual field. Specifically (see Fig. 5), sampling neurons along any sequence of sites starting near the V2d border, and moving rostrally, should reveal RFs that move from near the HM of the visual field toward the VM, in the lower half of the visual field.

**Fig. 6.**
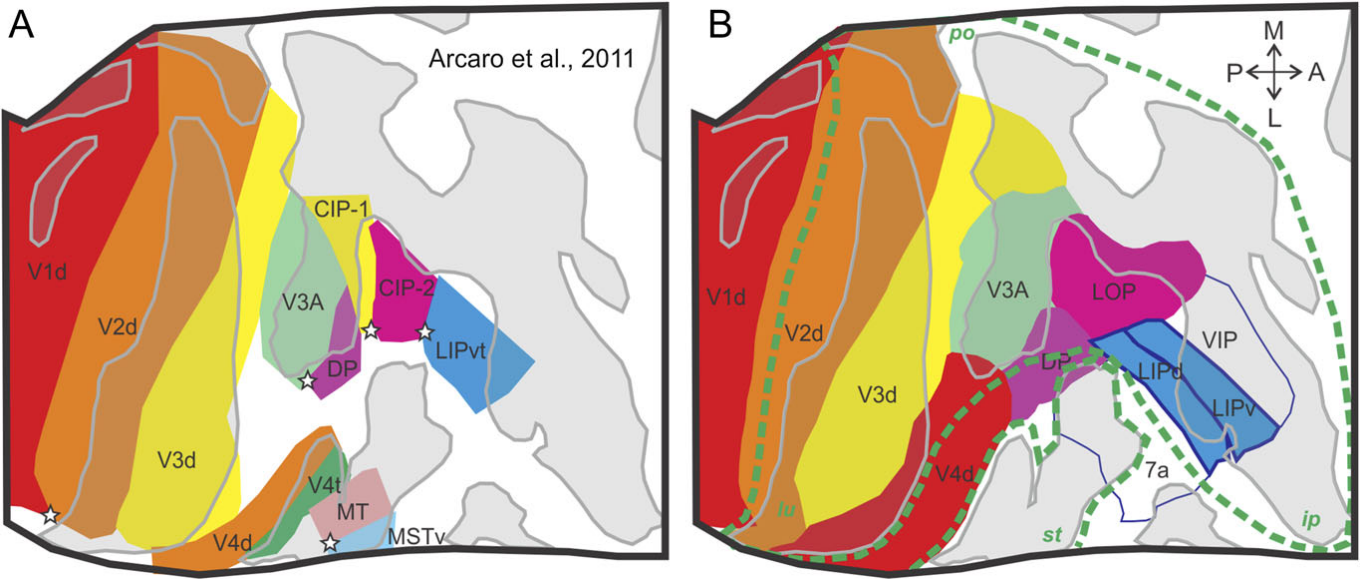
Functional imaging studies in the macaque reveal a continuous dorsal V3 anterior to dorsal V2. (**A**) Functional magnetic resonance imaging of the dorsal posterior cortex in the alert macaque monkey reveals the layout of the cortical regions anterior to V2. Note the continuous arrangement of V3d. Stars indicate the foveal representation. (**B**) Same as in (**A**), but derived from a combination of electrophysiological, connectivity, and myeloarchitectonic methods. Adapted from Van Essen et al., (2001). The border of the gray patches represents the points of lowest curvature on the cortex, with the midpoint across the dark gray region representing the fundus of the sulcal convexity. The green dashed lines in (**B**) indicate the border of the sulci. Regions delimited by the green dashed lines indicate the cortical areas, which are buried inside sulci, corresponding to the gray patches in Fig. 1B. Adapted from Arcaro et al., (2011).

Our scheme of V3, with both a V3v and V3d, finds support also in functional magnetic resonance imaging (FMRI) (Arcaro et al., 2011) and anatomical studies (Lyon & Kaas, 2002) in the macaque. Arcaro et al., (2011), using fMRI in the awake macaque trained to maintain fixation (Fig. 6), described a large-scale visuotopic organization along lateral portions of the posterior parietal cortex (PPC) anterior to area V3A, and extending into the lateral intraparietal sulcus (LIP). By considering both the polar angle and the eccentricity phase estimates, they identified four visuotopically-organized areas representing the contralateral visual field within the PPC. In the dorsal portions of the prelunate gyrus, they identified an area, DP that predominantly contained a representation of the lower visual field. The portions of DP located within the PO sulcus represented the horizontal and lower VMs. Fig. 6A depicts the organization of the dorsal visual areas in the macaque, as mapped by Arcaro et al. (2011). Note the correspondence with the map originally proposed by Van Essen et al. (2001), also in the macaque (Fig. 6B). Caution is needed when interpreting topographical studies carried out using fMRI methods. Topographical studies using electrophysiological methods in anesthetized monkeys benefit from the fact that eye movements in this kind of preparation can be pharmacologically abolished. In Arcaro et al. (2011), the authors went through the heroic task of training monkeys to fixate for several minutes in order to carry out their mapping in the awaken monkey. The downside is that their fixation window had to be enlarged to 4 deg by 4 deg diameter in order to accommodate for eye movement behavior. Variability in eye fixation, associated with the comparatively noisy nature of the fMRI signal gave rise in Arcaro et al. (2011) to maps with imprecise or ambiguous landmarks, such as the horizontal and VM representations, which are instrumental for areal delimitations. Overall, however, the fMRI results by Arcaro et al. (2011) are impressive and consistent with our electrophysiological recordings in V3d and V4d (Gattass et al., 1988). Additionally, they found area DP to represent mainly the contralateral lower visual field (Andersen et al., 1990). These results are also consistent with fMRI studies that have found representations of the lower visual field within the dorsal prelunate gyrus (Fize et al., 2003).

## Area V3 in the New World capuchin monkey

**Fig. 7.**
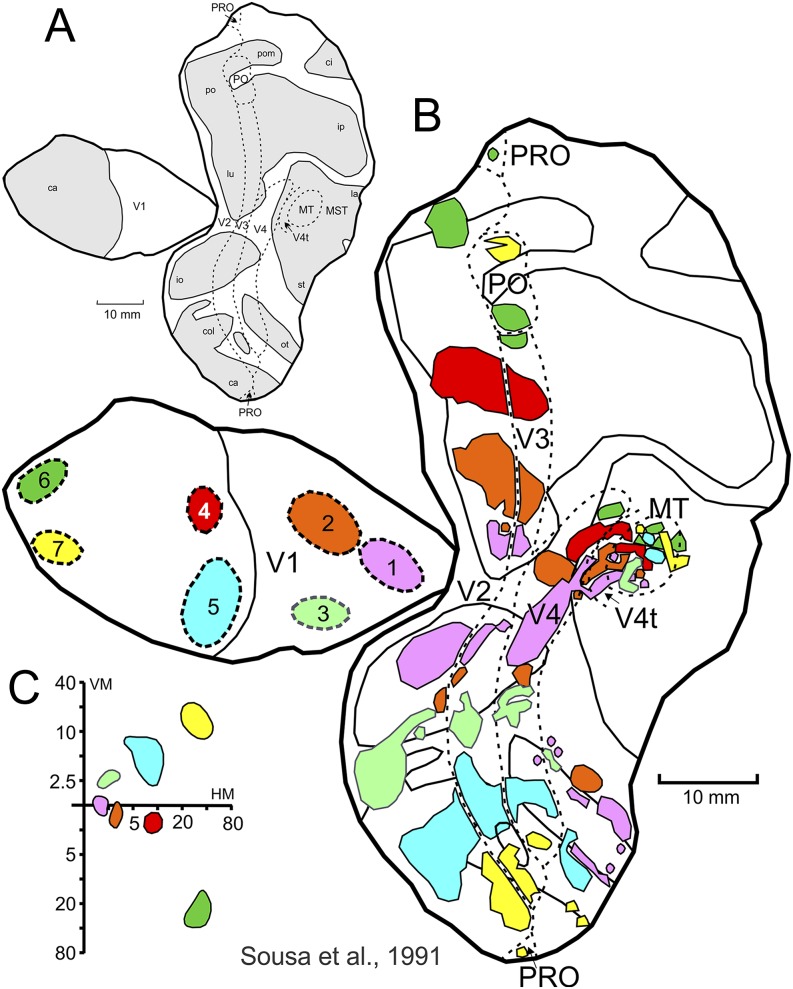
Extrastriate feedback projections to V1 indicate that ventral and dorsal V3 comprise a single homogeneous area, distinct from PO. (**A**) Flattened reconstruction of the posterior cerebral cortex similar to the illustration shown in Fig. 1B. (**B**) Flattened cortex exhibiting the injection sites (dotted outline blobs) located throughout area V1 (injections 1–7), along with their corresponding projection sites in the extrastriate cortex. Injection and projection sites are labeled with the same color to aid visualization. (**C**) Projection onto the contralateral visual hemifield of the location and extent of the seven injection sites. Thin lines in (**A**) and (**B**) indicate the lips of cortical sulci, while dotted lines indicate myeloarchitectonic borders of selected extrastriate visual areas. Refer to Fig. 1 for the corresponding topography of the various areas. Adapted from Sousa et al. (1991).

Anatomical connections of V1 and MT in the diurnal New World capuchin monkey suggested a lower field representation of V3 anterior to the lower field representation of V2, dorsally (Sousa et al., 1991; Rosa et al., 1993). In Fig. 7, we illustrate the results from retrograde tracer injections in V1. Note that the connectivity between V1 and V3 follows the same precise topographic pattern as the one observed between V1 and V2. Rosa et al. (2000) studied the visuotopic organization of the “third tier” visual cortex on the ventral surface of the occipital and posterior temporal lobes in the capuchin monkey. Unlike in the dorsal cortex anterior to V2, where there is evidence for multiple visual field representations, in both New World and Old World monkeys (for reviews, see Felleman & Van Essen, 1991; Kaas, 1997), the entire strip of cortex inserted between the ventral subdivisions of V2 and V4 formed a single, systematic representation of the upper contralateral quadrant. This region (for which we adopted the designation V3v) is also distinct from adjacent areas in terms of myeloarchitecture and neuronal RF size. With the exception of a slight invasion at the V2v/V3v border, no lower quadrant representation was observed in V3v or in the surrounding ventral cortex. Thus, two possibilities exist: either V3v is an area in itself that contains a representation limited to the upper half of the visual field (as suggested for Old World monkeys by Newsome et al., 1986), or V3v is part of a larger area that encompasses a representation of the lower quadrant in dorsal or dorsolateral extrastriate cortex. This lower quadrant representation could be continuous with the upper quadrant representation or be located in a segregated “island” of cortex, as suggested by Gattass et al. (1988) for the macaque (see Fig. 1).

Rosa et al. (2000) have argued against the idea that V3v represents the entire extent of a visual area in New World monkeys. The sector of the visual field represented in V3v largely excludes the lower quadrant, with the exception of occasional RFs mapped for cells located near the V2 border. These RFs, which cover only a few degrees below the HM, were assigned either to V2 or V3v on the basis of their size. There are no other lower quadrant representations adjacent to V3v in the ventral cortex. Thus, previous references to “a highly compressed representation of the lower quadrant” (Weller & Kaas, 1985; Burkhalter et al., 1986) appear to refer solely to the V2/V3v border region. The degree of invasion of the lower quadrant by RF centers of cells at the V2/V3v border is, in the vast majority of cases, within the margin of error introduced by the technique used for estimating the position of the fovea (0.5–1 deg) and was never found to exceed 2 deg. Moreover, in areas with a second-order representation, there is usually a slight overlap between the sectors of the visual field represented on each side of the field discontinuity (e.g., between the parts of the visual field represented in dorsal and ventral V2; see Rosa et al., 1988*b*, 1994, 1997, 2000). Thus, the existence of RFs that invade the lower visual field quadrant cannot be used as a valid argument in favor of a V3v as a “complete” area, representing both quadrants. In addition, the myeloarchitecture of the ventral and dorsolateral areas is similar (Rosa et al., 1993, 2000; Piñon et al., 1998). We therefore conclude that there is no compelling reason to view V3v in the capuchin monkey as an individual area restricted to the ventral cortex, or complete in terms of visual field representation. However, as pointed out by Rosa et al. (1993), it is still an open question as to how far V3d extends dorsally in the capuchin monkey.

## Continuous *versus* split representation of V3 fovea

Two proposals have been made for the organization of the V3 fovea. Initial work in the macaque by Zeki (1969) suggested that the dorsal and ventral portions of V3 would be segregated into two islands, with the gap coinciding with the foveal representation. The second proposal, based on studies in small New World Monkeys (Rosa et al., 2013), is that the entire V3 (i.e., VLP), including the fovea, is a continuous cortical segment (see Fig. 5). Defining the borders of a cortical area is a challenging task, particularly at the foveal representation and in regions buried inside sulci. The various techniques typically used to delimit areal borders (myeloarchitecture, electrophysiology, and connectivity) rely on the specific methodology being employed. For example, the angle of histological sectioning may influence the precision of border detection. Border detection based on myeloarchitecture is best achieved when the plane of sectioning is orthogonal to the border. Coronal sections are adequate for analyzing parafoveal and peripheral regions, while horizontal sections are best for analyzing central field representations. Parasagittal cuts transect the foveal region tangentially, which makes border detection difficult.

RF mapping using electrophysiological techniques has also proven useful. What we usually seek with sequential electrode penetrations are reversals in RF mapping progressions, often associated with changes in RF size and property. Electrophysiology has a resolution of approximately 0.3–1 mm when investigating cortical regions with high cortical magnification, such as the foveal representation. Thus, border detection becomes masked in places where V3 width narrows down to 1.5 or less. To address this challenge, we usually combine RF progression reversals with myeloarchitectonic transitions in order to draw the border between visual areas. In each histological section, the myeloarchitectonic transition zone usually ranges from 0.5 to 1 mm. We usually place the border at the center of the transition zone.

For the reasons stated above, we feel that V3 foveal organization cannot be definitely resolved with the currently available data. Our results hint that V3 in the macaque is divided into dorsal and ventral portions separated by an intrusion of foveal V4 (Gattass et al., 1988). This model for V3 organization is similar to that proposed by Zeki (1969). In capuchins, we found cases where V3 seemed continuous but narrowed down to a small strip of cortex at the foveal region (one case in Sousa et al., 1991). However, the most common arrangement was a V3 split into dorsal and ventral domains (Piñon et al., 1998).

## VP or V3: In conclusion

To summarize the present situation, there is widespread agreement that, in Old World monkeys, V3v has a strip-like shape with a systematic representation of the upper quadrant. The same seems to be true for small New World monkeys (Newsome & Allman, 1980; Rosa & Tweedale, 2000). Anatomical connections of V1 in the capuchin monkey (Sousa et al., 1991) revealed a lower field representation for area V3 anterior to the lower field representation of V2, dorsally. Nonetheless, Kaas (1997) has argued that V3v (VP) itself might contain a complete representation of the visual field, distinct from that found in dorsolateral and dorsal areas. This is an important issue for understanding the organization of the cortex anterior to V2 and the homologies between species. In particular, the demonstration of a substantial lower quadrant representation in V3v would support the distinction between this region and others located dorsal to it. This distinction has been questioned both by studies that propose that V3v continues dorsally, to include part of the dorsolateral cortex interposed between V2 and MT (Rosa, 1997; Rosa et al., 1997), and by those proposing that it is part of a much larger area which, like the “original V3”, includes the dorsal cortex anterior to V2 (Zeki 1969, 1977; Gattass et al., 1985, 1988).

There is currently no compelling reason for ruling out a dorsal extension of V3v, although the exact extent of this area remains the subject of further studies. Thus, it is likely that V3v will prove to be part of a larger visual area in the capuchin monkey. Why, then, have previous investigators concluded that V3v is an area restricted to the ventral cortex?

Early studies in both the macaque and owl monkey illustrate a V3v (or “VP”) that is bordered dorsally by V4 (or DL) (e.g., Newsome & Allman 1980; Van Essen, 1985). Neuroimaging studies in humans (e.g., Sereno et al., 1995; Tootell et al., 1997), preliminary electrophysiological observations in New World monkeys (Rosa et al., 1988*b*, 1997), and single-unit responses in macaques (Zeki 1983; Burkhalter & Van Essen, 1986) all converge to support the view that an area including V3v also encompasses a lower quadrant representation that extends at least to the dorsolateral cortex.

Recently, Lyon and Connolly (2012), in their review of V3, have concluded that evidence obtained in several primate species supports the hypothesis that an elongated V3 forming a complete map of the visual field occupies the region anterior to V2. One central prediction of Lyon & Connolly's scheme is that cells in the dorsal half of V3 will always have RFs representing the lower half of the visual field. However, Rosa et al. (2013) disagreed with the conclusions of Lyon and Connolly (2012) regarding the dorsal location and extent of V3. They argued that studies that mapped in detail the RFs (Allman & Kaas, 1975; Sereno et al., 1994; Rosa & Schmid, 1995) and anatomical connections (Rosa et al., 2009; Jeffs et al., 2013) of the cortex immediately rostral to the dorsal half of V2, particularly in small New World monkeys, demonstrated the existence of an upper field representation in this region, which they called the dorsomedial area (DM). According to this scheme, a V3-like area, which is less extensive than that proposed by Lyon and Connolly (2012), occupies only the lateral and ventral aspects of the third tier cortex, and is referred to as the ventrolateral posterior area (VLP) (Rosa & Manger, 2005). Electrophysiological recordings in the cortex adjacent to dorsal V2 in the marmoset and owl monkeys have consistently revealed RFs that drift toward the upper VM, as shown in Fig. 5. Indeed, there is no evidence for even a partial lower quadrant representation between V2 and DM, either in this sequence or in many others illustrated in previous studies of marmoset and owl monkeys (Allman & Kaas, 1975; Krubitzer & Kaas, 1993; Sereno et al., 1994; Rosa & Schmid, 1995; Rosa et al., 2009). In summary, the exact location and extent of the lower field representation of V3 remains controversial, with the possibility that lissencephalic (e.g., owl and marmoset monkeys) and gyrencephalic (e.g., rhesus and capuchin) monkeys vary to some extent in the configuration of this brain region.

## Area PO

Area PO was initially defined as a myeloarchitectonically distinct region, containing a complex visuotopic map with a de-emphasis of the central field representation (Covey et al., 1982; Gattass et al., 1985; Neuenschwander et al., 1994). In our first assessment of area PO (Covey et al., 1982; Gattass et al., 1985), we overestimate its size. The original description included two myeloarchitectonic areas that were later named areas PO and POd (Neuenschwander et al., 1994). The initial injections carried out by Colby et al. (1988) were restricted to the ventral portion of the anterior bank of the PO cleft, in the myeloarchitectonically homogeneous region we currently call PO. Thus, PO was initially defined as comprising the entire anterior bank of the PO cleft (Gattass et al., 1985). In a later review, we proposed that the anterior bank of the PO sulcus might be composed of more than one visual area (Gattass et al., 1985). The study of the connections and myeloarchitecture of the anterior bank of the PO sulcus revealed that area PO is located ventrally in the anterior bank and that it receives projections from the peripheral representation of both V1 and V2 (Colby et al., 1988).

Covey et al. (1982) proposed that area PO in the macaque, which borders V2 in the ventral portion of the PO cleft, is homologous to area M of the Aotus. In the Aotus, the medial visual area M is located within cytoarchitectonic area 19 and has a complete representation of the contralateral visual field, with little emphasis on the representation of central vision. Area M borders V2 anteriorly and shares with it part of the HM representation. A similar arrangement has also been described for the macaque (Covey et al., 1982), where area PO was included within cytoarchitectonic area OA of von Bonin and Bailey (1947).

## Borders of areas PO and POd

**Fig. 8.**
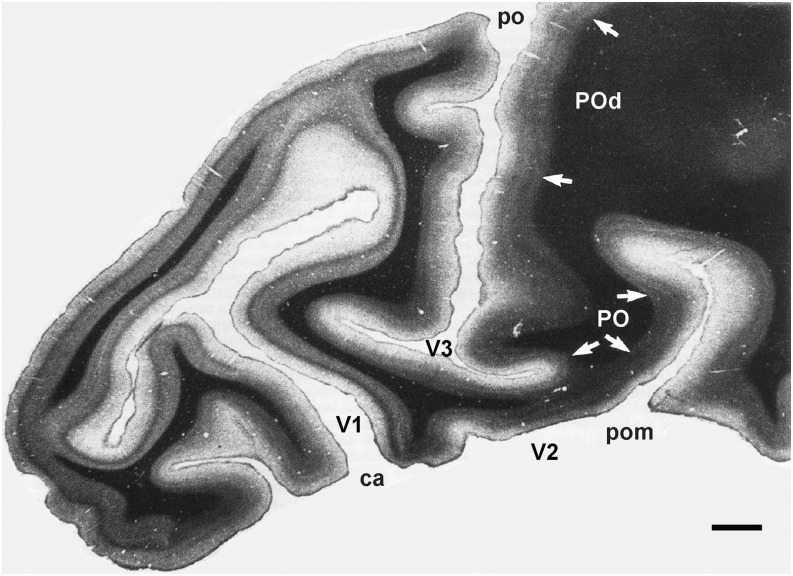
Myeloarchitecture delimits the borders of areas V3, PO and POd. See text for details. Gallyas' stained parasagittal section from the same animal described in Colby et al. (1988).

In order to precisely determine the boundaries of areas PO and POd, Neuenschwander et al. (1994) studied the correspondence between electrophysiological data and myeloarchitectonic transitions in the parietal cortex of the capuchin monkey. In addition, connectional data in the capuchin also supported the subdivision of the rostral bank of the PO sulcus into dorsal (POd) and ventral (PO) areas (Colby et al., 1988; Sousa et al., 1991; Rosa et al., 1993). The myeloarchitectonic pattern of PO corresponds to the conjunct-striated pattern described by Sanides (1972). Several myeloarchitectonic features are apparent when viewing a histological section of the PO region stained with the Gallyas' method (see Fig. 8). The infragranular layers of PO are heavily myelinated, the inner and outer bands of Baillarger are confluent, and the layers above the outer band of Baillarger are pale and less myelinated than those of the surrounding cortex. Myeloarchitectonic transitions between PO and the laterally and medially located areas are easily determined in coronal sections, while transitions between PO and the ventral and dorsal areas are better determined in parasagittal sections. The border of PO with POd coincides with a decrease in myelination in the infragranular layers. POd is less myelinated than PO and presents a more differentiated and less dense outer band of Baillarger. Posteriorly, PO is bordered by V2, an area that has a homogeneous pattern of myelination in the infragranular layers (Rosa et al., 1988*b*) but not as dense as that of PO. In addition, the outer band of Baillarger in V2 is less conspicuous, and less dense than those of PO and POm. In some animals, the lateral portion of PO has an approximately 1.5 mm wide myeloarchitectonic transitional zone, which borders area V3d. This transition zone is less dense than the core of PO, but it is more myelinated than V3d. Based on topographic data, the representation of the visual field in this transition zone complements that of the core of PO and was therefore considered as an integral part of PO. In other animals, no myeloarchitectonic transitional zone was observed, and the V3d/PO border was therefore not clear in all sections. The dorsal border of PO with POd is usually determined in parasagittal sections. POd is less myelinated than PO and presents individualized inner and outer bands of Baillarger (Fig. 8). The inner band gradually joins the white matter. The outer band is thinner than that of PO but well differentiated.

We used the procedure described by Maunsell and Van Essen (1987) to assess which portions of the visual field are over-represented in areas PO and POd, as well as to determine their visuotopic orderliness. In brief, this procedure consists in back-transforming onto the visual field a grid defined by an array of interpolated coordinates corresponding to equally spaced points in the cortex. For the case of a precisely organized cortical map, a square grid on the cortex would translate into an orderly cobweb pattern in the visual field (Schwartz, 1980; Van Essen et al., 1984; Maunsell & Van Essen, 1987; Fiorani et al., 1989). This analysis showed interanimal variability regarding the degree of visuotopic orderliness in PO and POd. We observed a considerable organization along the isopolar dimension, and a more irregular organization along the isoeccentric dimension (Fig. 2). The back-transformed maps reported by Neunschwander et al. (1994) suggest a greater representation of the visual field region ranging from 20–50 deg eccentricity. Area PO also resembles area M in having a limited central vision representation. We observed no RF centers with eccentricity values smaller than 15 deg. For this reason, our visuotopic map of area PO (Fig. 2) does not contain a representation of the central field. However, this result does not imply that the central field is not represented in PO in as much as some of the RFs do include the fovea.

The topographical peculiarities of areas PO and POd were evident in the back-transformed maps we generated using our electrophysiological data. Our assumption that PO is an area distinct from POd was based primarily on the topographic analysis performed for one animal we studied. We considered the multiplicities of central and peripheral field representations as evidence for the existence of two distinct visual areas, each containing a virtually complete representation of the visual hemifield. Neunschwander et al. (1994) showed back-transformed grids (“webs”) obtained for PO and POd that superimposed on each other in the visual field. In one case, an unquestionable re-representation was observed in area POd, defined initially on myeloarchitectonic grounds. Likewise, the dorsal portion of PO contained a re-representation of a peripheral portion of the visual field. One could interpret these data as a result of irregularities due to the complex representation of the visual field in PO and, thus, equivalent to the local representations found, for example, in area V2 (Rosa et al., 1988*b*). We do not favor this hypothesis inasmuch as the representations shown in the back-transformed grids in each case were comparable to those of the back-transformed maps of all other cases we studied.

## A case for PO as a unique cortical area

Connectivity studies by Van Essen et al., 1986 were the first to suggest that PO constituted the peripheral representation of V3d. Particularly, they found that layer 4B of V1 projected to V3d and PO, but not to V3v, which hinted that V3d and PO might constitute a single area, distinct from V3v.

In addition to our myeloarchitonic data (see above), here we also review some of our connectivity studies, where we used densely spaced tracer injections in the macaque and capuchin monkeys, to make the case that area PO constitutes a single and distinct visual area. Particularly, we argue that PO is not a part of V3d, where it would represent the peripheral portion of the visual field, nor that PO is part of a larger area V6, as proposed by Galletti et al. (1999*a*, 2001). In Fig. 3A, we reviewed data from tracer injections in V2 that served to delineate the extent of V3v and V3d in the macaque monkey. The same tracer injections, when carried out in the more peripheral portions of V2 (eccentricities of 30 deg and greater), are also capable of revealing the extent of PO and its border with V3d. Take, for example, injections number 5, 6, and 11 in Fig. 3A, all placed in the peripheral representation of V2. Note that, injection 5 gives rise to two projection sites in the medial-dorsal cortex anterior to V2d (i.e., two green blobs; one attributed to V3d and the other to PO). Additionally, injection 6, which was placed at a more peripheral representation in V2d, shows a projection site which is more lateral in PO compared to the projection of injection 5 (i.e., olive blob in between the two green blobs). If it were indeed the case that PO constitutes the peripheral representation of V3d, then only one projection site should have been observed for injection 5, and injection 6 should have been located more medially, obeying the orderly progression of isoeccentricity lines for V3d. On the contrary, the projection site of injection 6 labels both V3d and PO, along their border region, as it would be expected for projections representing the far periphery in these areas. Injection 11, which was placed at the peripheral representation of V2v labels the medial region of PO (i.e., yellow blob), also as expected from the topography we propose (Covey et al., 1982; Gattass et al., 1985; Colby et al., 1988; Neuenschwander et al., 1994, see Fig. 1).

**Fig. 9.**
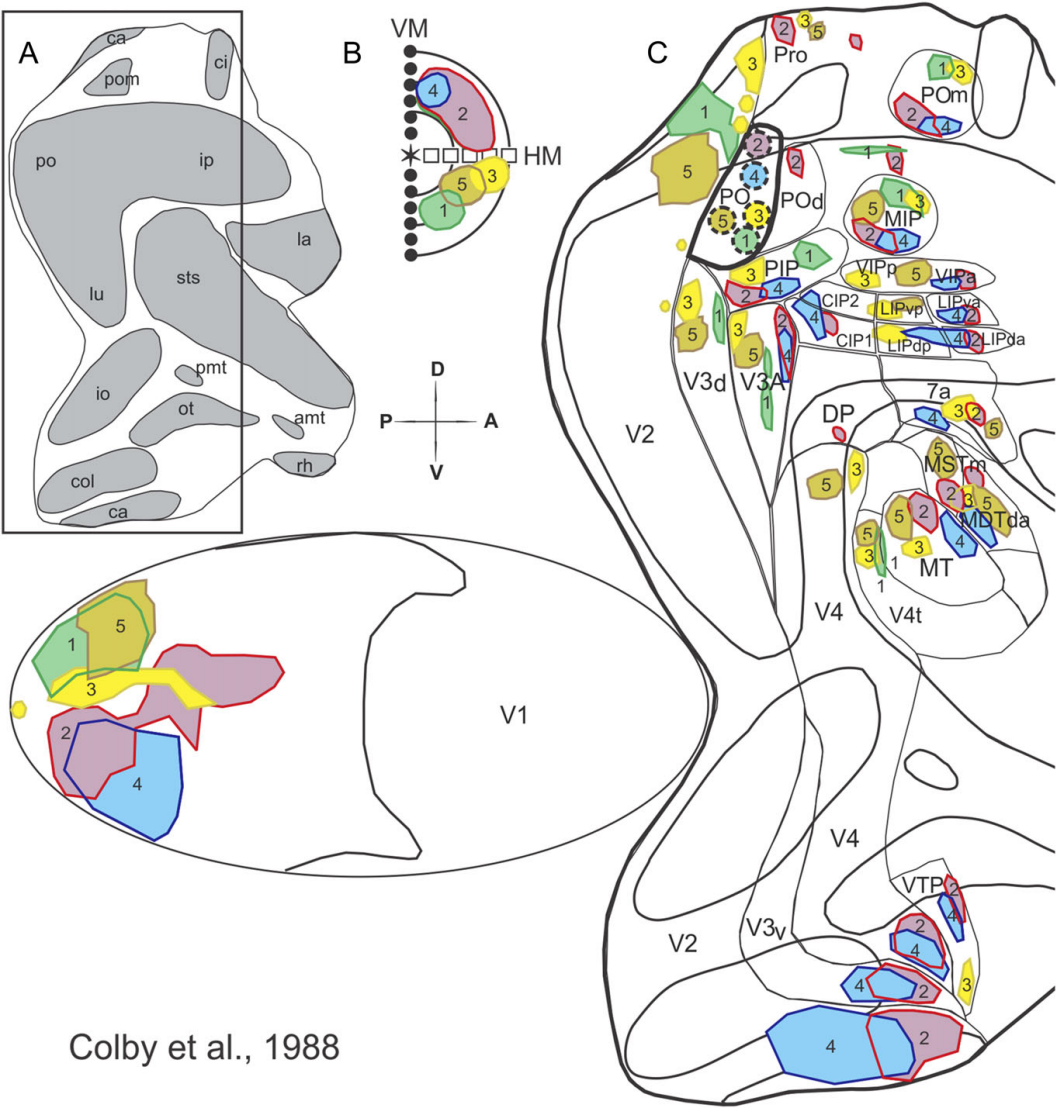
Feedforward projections to PO support its status as a single cortical area, distinct from V3d. (**A**) Illustration of a flattened cortex equivalent to the one shown in Fig. 1A. The rectangle delimits the extrastriate cortical region depicted in (**C**). (**B**) Projections onto the contralateral visual field of the location and extent of the five PO injections. (**C**) Injection sites in PO (dashed outline circles, numbered 1–5) and their corresponding projection sites in striate and extrastriate cortex. Injection and projection sites are labeled with the same color to aid visualization. A, anterior; P, posterior; D, dorsal and V, ventral. Adapted from Colby et al. (1988).

The above findings are corroborated by tracer injections in PO, also in the macaque. Fig. 9 shows feedforward projections from the lower and upper field representations of the far periphery of V1, V2, and V3 to PO (Colby et al., 1988). Specifically, injections 1, 3, and 5 project to neighboring V3d, emphasizing that PO does not constitute the peripheral representation of V3d, but rather a unique cortical area. The other nearby injections in PO (2 and 4) project to the far periphery upper field representation of V2, V3 and V4 (ventral cortex), also as predicted from our proposed topography for PO. Note the lack of organization based on isoeccentricity lines, despite the fact that the upper *versus* lower polarity of hemifield representations is still preserved (e.g., medial injections in PO project exclusively to ventral V2, V3, and V4; see red and blue blobs in Fig. 9). Notably, all the five injections shown in Fig. 9 occupy the entire extent of PO. The resulting projection sites label exclusively the peripheral visual field representation in the surrounding cortex, leaving little or no room in which the fovea could be represented. We believe that these results are incompatible with the proposal put forward by Galletti et al. (1999*a*, 2001), where PO would be part of a larger area V6. Finally, note that PO is connected to other topographically organized areas in the parietal cortex, designated here as POm and MIP.

Altogether, our connectivity studies disagree with the initial finding presented by Van Essen et al., 1986. First, we found that both ventral and dorsal V1 and V3 send projections to PO (Colby et al., 1988; see Fig. 9). These projections obey the upper *versus* lower polarity of visual field representation. The V1 projecting neurons were located both in layer 4B and in the supragranular layers. The V3 projecting neurons, both in its ventral and dorsal segments, were concentrated in the supragranular layers. Second, both V3d and V3v send feedback projections to V1 (Sousa et al., 1991, see Fig. 7), suggesting that they are a homogeneous visual area. Despite the fact that we did not study the projections from V1 to V3, we assume that feedforward and feedback projections between early visual areas are generally symmetrical.

Adding to the diversity in nomenclature, Galletti et al. (1996, 1999*a*) have proposed the name of “V6” to visual area PO of the macaque. Based on visual topography, RF size and myeloarchitecture, we showed two distinct areas in the anterior bank of PO sulcus of the capuchin monkey, and named them PO and POd (Neuenschwander et al., 1994). POd has larger RFs and a distinct lighter myeloarchitecture. More recently, POd was renamed as “V6A” by Galletti et al. (1999*b*, 2001).

Area V6 of Galletti et al. (1999*a*, 2001) is roughly at the same cortical location as the one we reported for PO (Colby et al., 1988; Neuenschwander et al., 1994). However, V6 and PO exhibit very different topographic organizations: (1) area V6 is described as having a central field representation, with the fovea located laterally. We do not observe any fovea representation in PO; (2) V6 is organized in isoeccentric and isopolar lines, while PO shows no clear organization in the isoeccentric domain; (3) the representation of V6 upper visual field is more medial than that described for area PO.

Altogether, the organization of V6 lower visual field resembles that of area DM described in the marmoset (Rosa et al., 2013), despite the fact that their reported organizations do not suggest they are homologous. Therefore, there is no evidence to support the notion that V6, as described by Galletti et al. (1999*a*, 2001), corresponds to area PO that we have described in the macaque and capuchin monkeys (Colby et al., 1988; Neuenschwander et al., 1994).

## Comparisons with small New World monkeys

Discussions regarding the nomenclature of cortical areas are especially useful in a review article such as this one. Grasping the homologies across species has been hindered by the variety of terms and acronyms often used to designate the same visual area. Area MT, for example, was first described as a fully defined visual area in the owl monkey (Allman & Kaas, 1971), and shortly after in the Galagos (Allman et al., 1973). Therefore, when we first described the visual topography of the striate recipient area located in the superior temporal sulcus of the macaque (Gattass & Gross, 1981), we chose to designate it area MT in an effort to standardize the nomenclature used in the literature. Zeki (1980) also used the term MT when studying the corresponding visual area in the owl monkey. Surprisingly, however, he did not acknowledge that MT and the macaque movement area (V5) correspond to homologous regions in the two species.

Fig. 5 summarizes a comparative view for the organization of the posterior cortical visual areas in Old World (macaque) and New World (marmoset) monkeys. Despite the fact that the capuchin is a New World monkey, our results indicate that its cortical organization is much more closely related to the macaque than to the marmoset. The recent review by Lyon and Connolly (2012) suggests that in the macaque an elongated V3 is located anterior to V2, forming a complete map of the visual field. This view supports our original proposal based on electrophysiology (Gattass et al., 1988; Piñon et al., 1998) and anatomy (Gattass et al., 1997; Ungerleider et al., 2008). It is also confirmed by a recent study of the topographic organization of dorsal extrastriate cortex using fMRI and phase-encoded retinotopic mapping in monkeys (Arcaro et al., 2011). These results, which are shown in Fig. 6, corroborate the notion that V3d is a continuous region spanning a large extent of V2's anterior border. This organization differs from that proposed for the marmoset (Rosa et al., 2013) where the dorsal portion of VLP (V3) swings forward, thereby allowing DA and DM to border V2 medially. The study where we injected retrograde tracers in area V1 (Sousa et al., 1991, see Fig. 7) clearly shows that the areal layout found in the capuchin monkey is similar to the one found in the macaque. Note in Fig. 7 that V1 receives orderly projections from a continuous segment of area V2. V1 also receives projections from another continuous strip of cortex located anterior to V2, namely area V3. We did not observe any swinging forward of V3 in the capuchin, contrary to the organization described for VLP (the homolog of area V3) in the marmoset (Rosa et al., 2013). Additionally, there is evidence that V3d in the capuchin borders area PO, medially. Note that injection site #7 (located at V1 periphery, upper hemifield representation) in Fig. 7 labels areas V2v and V3v but also area PO, which is located dorsally. The complementary injection site #6 (located at V1 periphery, lower hemifield representation) projects to the peripheral representation of V2d but also to area PO. A DM-like organization would have predicted projection site 7 (yellow) to be located more medially compared to projection site 6 (green). Finally, the cluster of labeled cells is contiguous, with no evidence for an intervening visual area between V2d and PO.

In our view, V3 in the macaque and capuchin monkeys is homologous to VLP in the marmoset, while PO is homologous to area M and not area DM, as it has been previously proposed by Rosa et al. (2013). The following pieces of evidence support our claim: (1) both PO and area M emphasize the peripheral, as compared to the foveal representation, while DM has a clear representation of the central vision; (2) DM has a clear representation of the isoeccentricity lines, while PO does not; (3) PO and DM have distinct myeloarchitecture (Colby et al., 1988; Sousa et al., 1991; Neuenschwander et al., 1994; Rosa et al., 2013).

We are convinced that the topography of the areas anterior to V2 varies not only across primate species but also across individuals within the same species (Gattass et al., 1988; Piñon et al., 1998). Dorsal V3, for example, is continuous and borders area PO medially in most animals. In some cases, however, area PIP can stand in-between V3d and PO (Colby et al., 1988). The reasons for this diversity are still unclear. We think that future work should also address the topographic organization of recently described areas located in the caudal region of the intraparietal sulcus, such as CIP-1 and CIP-2. Specifically, we think that systematic studies using multiple electrode arrays and automatic RF mapping methods will shed new light into the functional organization of areas PO, POd, as well as describe new areas located in the annectent gyrus and in the intraparietal cortex.

## Abbreviations

### Cortical visual areas

CIP-1: caudal intraparietal area 1
CIP-2: caudal intraparietal area 2
DP: dorsal posterior area of the pre-lunate gyrus
FST: visual area FST
LIPd: dorsal portion of lateral intraparietal area
LIPv: ventral portion of lateral intraparietal area
MIP: medial intraparietal area
MST: medial superior temporal area
MT: visual area MT
MTp: peripheral portion of MT
PIP: posterior intraparietal area
PO: parieto-occipital area
Pod: dorsal parieto-occipital area
PRO: prostriate area
Tea: anterior portion of area TE
Tem: medial portion of area TE
TEO: posterior inferior temporal cortex
TEp: posterior portion of area TE
TH: cytoarchitectonic area TH
V1: primary visual cortex
V2: visual area 2
V3A: visual complex V3, part A
V3d: dorsal portion of visual area 3
V3v: ventral portion of visual area 3
V4: visual area 4
V4t: V4 transition zone
VIP: ventral intraparietal area
VTF: visual portion of parahippocampal TF

### Cortical sulci

amt: anterior middle temporal sulcus
ar: arcuate sulcus
ca: calcarine fissure
ce: central sulcus
ci: cingulate sulcus
co: collateral sulcus
ec: external calcarine sulcus
io: inferior occipital sulcus
ip: intraparietal sulcus
la: lateral sulcus
lu: lunate sulcus
orb: orbital sulcus
ot: occipitotemporal sulcus
p: principal sulcus
pmt: posterior middle temporal sulcus
po: parieto-occipital cleft
pom: medial parieto-occipital sulcus
rh: rhinal sulcus
sp: subparietal sulcus
st: superior temporal sulcus
